# Sound intensity-dependent cortical activation: implications of the electrical and vascular activity on auditory intensity

**DOI:** 10.1007/s11571-025-10281-7

**Published:** 2025-06-09

**Authors:** Vanesa Muñoz, Brenda Y. Angulo-Ruiz, Carlos M. Gómez

**Affiliations:** Human Psychobiology Laboratory, Experimental Psychology Department, University of Sevilla, Seville, Spain

**Keywords:** Auditory intensity, AEPs, FNIRS, Neurovascular coupling

## Abstract

Recent studies combining electroencephalography (EEG) and functional near-infrared spectroscopy (fNIRS) have shown promising results linking neural and vascular responses. This study analyzes the topographical effect of auditory stimulus intensity on cortical activation and explores neurovascular coupling between fNIRS hemodynamic signals and auditory-evoked potentials (AEPs), extracted from EEG. Forty healthy volunteers (13 males, 27 females; mean age = 22.27 ± 3.96 years) listened to complex tones of varying intensities (50-, 70-, and 90-dB SPL) across seven frequencies (range of 400–2750 Hz) in blocks of five, while EEG and fNIRS were recorded. PERMANOVA analysis revealed that increasing intensity modulated hemodynamic activity, leading to amplitude changes and enhanced recruitment of auditory and prefrontal cortices. To isolate stimulus-specific activity, Spearman correlations were computed on residuals—components of AEPs and fNIRS responses with individual trends removed. The N1 amplitude increase was correlated with higher superior temporal gyrus (STG) and superior frontal gyrus (SFG) activity, and reduced activity in inferior frontal gyrus (IFG) for the oxygenated hemoglobin (HbO), while the deoxygenated hemoglobin (HbR) was associated with increased activity in one channel near the Supramarginal Gyrus (SMG). P2 amplitude increase was associated with higher activation in SFG and IFG for HbO, while for HbR with the activity in SMG, angular gyrus (AnG), SFG, and IFG. Additionally, internal correlations between fNIRS channels revealed strong associations within auditory and frontal regions. These findings provide insights into existing models of neurovascular coupling by showing how stimulus properties, such as intensity, modulate the relationship between neural activity and vascular responses.

**Supplementary Information:**

The online version contains supplementary material available at 10.1007/s11571-025-10281-7.

## Introduction

Functional near-infrared spectroscopy (fNIRS) is a non-invasive optical neuroimaging technique that measures hemodynamic changes following neural activation by quantifying changes in oxygenation and deoxygenation of hemoglobin. Neuronal activity is associated with increased vasodilation of cerebral arteries and with an excess of cerebral blood flow and volume (functional hyperemia). The amount of oxygen delivered to the activated brain region is greater than the rate at which it is consumed, resulting in an increase in oxygenated hemoglobin (HbO) and a decrease in deoxygenated hemoglobin (HbR), producing a typical response known as the cerebral hemodynamic response (Pinti et al. 2020). This physiological change is related to the concept of neurovascular coupling, which implies a coupling between neuronal activation in a given region and its subsequent oversupply of oxygen (Schei et al. 2012). As brain activity has been associated with changes in the optical properties of brain tissue, by the absorption and scattering properties of near-infrared light passing through the scalp, optical methods can provide a quantification of the hemodynamic variables following neural activation (Ferrari and Quaresima 2012). Thus, fNIRS devices use light sources, typically in the 650–1000 nm range, and detectors to capture changes in the non-absorbed light passing through brain tissue.

One of the main advantages of fNIRS is its tolerance to motion artifacts and the absence of scanner noise, making it a suitable alternative to functional magnetic resonance imaging (fMRI) for studying auditory processing. Some studies suggest that the fNIRS technique could be a good way to assess the auditory activation near Hesch’s gyrus (Shader et al. 2021), and to distinguish activation patterns between auditory and visual stimuli (Chen et al. 2015). In this context, has been suggested that the hemodynamic response to auditory stimuli may vary depending on intrinsic stimulus characteristics, such as duration (Zhang et al. 2022), frequency (Weiss et al. 2008), or intensity (Bauernfeind et al. 2018; Weder et al. 2018, 2020; Muñoz-Caracuel et al. 2021; Muñoz et al 2022, 2023), and even individual sensitivity (Steinmetzger et al. 2020).

Regarding the effect of sound intensity, some fNIRS studies have reported intensity-dependent activity in areas near the superior temporal gyrus (STG; Weder et al. 2018, 2020), and frontal regions (Bauernfeind et al. 2018; Muñoz et al. 2023). However, other studies have not observed this intensity-dependent effect (Chen et al. 2015; Muñoz-Caracuel et al. 2021; Muñoz et al. 2022). Two main limitations have been proposed: (i) the spatial limitation of fNIRS to the primary auditory cortex, which seems to be more sensitive to the perceived intensity (loudness) than to sound pressure level (SPL; Chen et al. 2015; Weder et al. 2020), and (ii), the physiological noise, especially when very high intensities are used, which could cause vasoconstriction and lead to false negatives (Muñoz-Caracuel et al. 2021; Muñoz et al. 2022). Although conflicting results have been obtained with the fNIRS technique, fMRI studies have strongly suggested the existence of a linear blood-oxygen-level-dependent (BOLD) response modulated by sound intensity in auditory cortical and subcortical areas (Brechmann et al. 2002; Hart et al. 2003; Sigalovsky and Melcher 2006) and have also included the discussion of the perceived loudness as the main predictor of activation in the primary auditory cortex (Hall et al. 2001; Langers et al. 2007; Röhl and Uppenkamp 2012).

In parallel, electroencephalography (EEG) studies have extensively documented intensity-dependent modulation of auditory evoked potentials (AEPs), particularly in P1, N1, and P2 components, as well as in the P2-N1 difference (Hegerl and Juckel 1993; Hegerl et al. 2001; Paiva et al. 2016). This phenomenon, known as intensity-dependent amplitude potentials (IDAP) or loudness dependence of auditory evoked potentials (LDAEP), has clinical relevance in affective disorders such as depression and bipolar disorder, where diagnosed subjects show a pronounced increase of AEPs amplitude with increasing intensity, indicating low serotonergic neurotransmission. This statement is supported by the fact that serotonin agonists reduce intensity dependence (Gallinat et al. 2000; Linka et al. 2005; Park and Lee 2013; Yoon et al. 2021; Fitzgerald 2024).

The primary auditory cortex, particularly layer IV, has been proposed as the main neural source of IDAP due to its serotonergic innervation (Hegerl and Juckel 1993; Hegerl et al. 2001). Similarly, the orbitofrontal cortex (OFC), which receives serotonergic projections, has been also implicated in IDAP, as increased fMRI signal intensity in the OFC has been linked to impulsivity (Mavrogiorgou et al. 2017). In contrast, in the study of the neural sources of AEPs, the sequence of P1-N1-P2 components has been associated with distinct neural generators, as identified through EEG and its counterpart, magnetoencephalography. On one hand, P1 has been primarily localized in the auditory cortex (Woldorff et al. 1993). On the other hand, the N1 component has been related to five different sources (reviewed by Atienza et al. 2001): (i) bilaterally in the supratemporal auditory cortices, (ii) the lateral surface of the temporal lobes, and (iii) the frontal motor and premotor cortices. Additionally, two frontal sources have been described: (iv) an obligatory component and (v) one that emerges only for interstimulus intervals longer than 4 s. The neural origins of P2 remain a topic of debate. Although some studies have proposed that P2 shares a frontocentral scalp distribution similar to N1 (Alcaini et al. 1994; Hyde 1997), other evidence suggests that the auditory source of P2 differs from that of N1 (Ross and Tremblay 2009). Further supporting frontal contribution, studies combining AEPs and fNIRS recordings have also highlighted frontal involvement in P2 generation (Muñoz et al. 2023). Combined EEG-fMRI studies support an auditory source for IDAP, demonstrating strong correlations between EEG source localization and BOLD responses in auditory regions when different sound intensities are presented (Mulert et al. 2005). However, fMRI research has also shown that sound intensity modulates activity not only within the auditory cortex (including Heschl’s gyrus) but also in other cortical areas at higher intensities (90–100 dB), such as the insular and opercular cortices, as well as the orbitofrontal cortex (Thaerig et al. 2008; Neuner et al. 2014).

To our knowledge, there are no studies in the literature that analyze changes in auditory stimulus intensity and their neural sources using fNIRS and EEG at a topographical level. While EEG remains a gold standard for measuring AEPs, it has limitations in spatial resolution, particularly when high-density array methods are not feasible due to time, cost, or participant constraints. Furthermore, although source localization EEG can improve spatial precision, it often relies on inverse solutions with inherent uncertainties. In contrast, fNIRS provides a direct measure of localized hemodynamic responses with better sensitivity to cortical topography, particularly in superficial brain regions. Combining EEG and fNIRS in a single setup is also comfortable for participants, as both systems can be integrated into one cap. Moreover, this integration may reduce preparation time, especially given that EEG setup can be lengthy depending on the type and number of electrodes used. In this sense, incorporating fNIRS alongside EEG could allow for a reduction in the number of electrodes without compromising the detection of stimulus-related effects. Finally, neural electrical activity and brain blood flow although interacting entities have their regulatory systems, and the simultaneous recording of both would give complementary insights into brain function. Therefore, this dual-modality could provide a more efficient and practical way to study brain responses to different stimulus intensities, particularly in sensitive populations such as children or clinical groups, where shorter setup times, reduced discomfort, and increased tolerance are critical.

In a previous study conducted in our laboratory (Muñoz et al. 2023) we found a correlation between the N1 component and the deoxygenated hemodynamic response in the left auditory cortex, and the P2 component with deoxygenated hemodynamic response in the right dorsolateral cortex. However, this study lacked the spatial resolution necessary to capture fine-grained topographical information. The present study aims to analyze neurovascular coupling by exploring whether auditory intensity modulates the hemodynamic response similar to its known effects on AEPs. Specifically, we aim to determine how electrical and hemodynamic responses align across brain regions measured with fNIRS. Thus, we seek to clarify whether the intensity-dependent dynamics of AEPs are reflected in vascular activity and to identify patterns of correspondence between these modalities. To address this, the present study analyzes the topographical effect of intensity on cortical activation using fNIRS alongside AEPs. By applying Spearman correlations on the residuals, we aimed to identify functional relationships between hemodynamic responses and AEP components, uncovering patterns of intensity-dependent neurovascular dynamics. This approach allows us to capture more specific aspects of how neural and vascular responses interact in auditory and prefrontal regions, isolating relationships beyond individual effects.

We hypothesized that lower-intensity stimuli would primarily activate the auditory cortices, with activation extending into prefrontal cortices as stimulus intensity increases, as has been analyzed by fMRI (Hall et al. 2001; Neuner et al. 2014). Furthermore, consistent with our previous findings (Muñoz et al. 2023), we expect distinct relationships between AEP components and cortical activation patterns: N1 should primarily align with auditory cortex activity, while P2 should be more prominent in prefrontal areas. By advancing the characterization of intensity-related neurovascular coupling, this study contributes not only to basic auditory neuroscience but also to the development of multimodal tools that could support future clinical and developmental research—especially in populations where fMRI or complex setups are not suitable or practical.

## Methods

### Participants

Forty volunteers (13 males and 27 females, mean age = 22.27 ± 3.96 years) participated in the study. As a selection criterion, all participants had normal hearing, and none of them had a history of neurological or psychiatric disorders. All but two of the participants were self-reported right-handed. Before the study, they were informed about the procedures and the experimental protocol, and then they signed an informed consent form. Prior to the experiment, all participants completed the Hearing Screening Inventory (Coren and Hakstian 1992) and all scored below 29 (mean = 21.5 ± 2.95).

### Experimental design

Previous to the experiment, a sound level meter (Tacklife SLM01, Dongguan Xingji Electronics Co) was used to check the SPL of the delivered stimuli, by measuring in each ear on the position of the subject the three intensity tones (60 trials, 20 of each intensity). The means for each intensity were intensity 1 = 48.9, intensity 2 = 72.6, and intensity 3 = 89.4. Given the device’s accuracy threshold of ± 1.5 dB, the intensities in the present study are referred to as 50-, 70-, and 90-dB SPL, respectively.

**Fig. 1:**
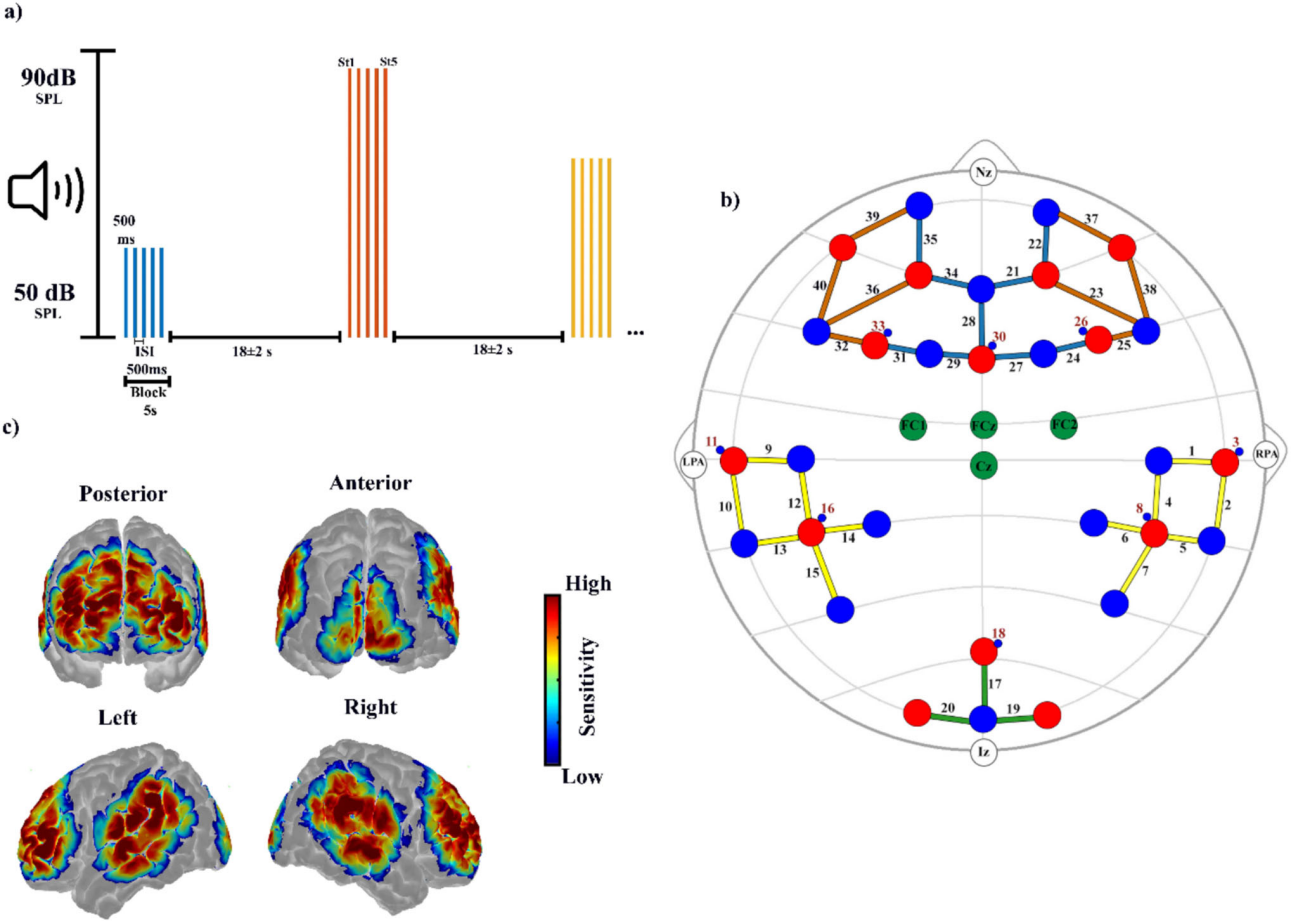
**a** Experimental protocol of the presented auditory stimulation. **b** Setup of fNIRS optodes and EEG electrodes; sources are shown in red, detectors in blue and EEG electrodes in green. The numbers correspond to the channel number (short channels in red font) and the lines show the channels formed by the optodes (auditory cortex: yellow; visual cortex: Green; Brodmann area 9-SFG: blue; Brodman area 45–47-IFG: orange). **c** Sensitivity maps created with AtlasViewer showing the coverage and depth of the fNIRS montage. ISI: Inter-Stimulus Interval, SPL: Sound Pressure Level

For the experimental section, we used complex tones (frequency modulated) composed by 7 frequencies (400, 850, 1150, 1650, 1950, 2450, 2750 Hz). The frequencies were calculated by multiplying the first frequency by 2 to 7 and then subtracting or adding 50 Hz to avoid harmonics. This type of tone was used because complex tones have been suggested to induce greater cortical activation than pure tones (Hall et al. 2001). In the recording section, a series of 5 stimuli (ST1-ST5) with a duration of 500 ms and an ISI of 500 ms were presented, each block of stimuli was delivered 20 times, for each intensity (50-, 70-, and 90-dB SPL), and were followed by 18 ± 2 s of silence (Fig. 1a), to allow to the hemodynamic response to return to baseline. Tones were presented completely randomly to each subject using the PsychoPy software. Participants were instructed to move as little as possible and to watch a silent movie while the tones were played. The movie was used as a distraction and no active responses were required. The session lasted approximately 25 min.

### Signal acquisition and processing

#### EEG

The EEG was recorded with a Brain Vision V-Amp DC amplifier (Brain Products, Munich, Germany) using active electrodes (ActiCAP) placed in the central position of the scalp (FCz, FC1, FC2, and Cz, Fig. 1b), these electrodes were selected by its known high amplitude for AEPs. Three additional electrodes to record eye movements were introduced: Fpz for vertical movements and F9 and F10 for horizontal movements. The reference was placed in the electrode PO10. The electrode impedance was kept below 20 kΩ. The DC amplification gain was 20,000, and the sampling rate was 1000 Hz. BrainVision Recorder 1.2 (Brain Products) was used for data acquisition.

EEG data were imported into EEGLAB v2021.1 (Swartz Center for Computational Neuroscience; Delorme and Makeig 2004) and MATLAB R2019b (MathWorks, Natick, MA, USA) software packages for the preprocessing step. The EEG data were band-pass filtered at 0.05–40 Hz, epoched (−3 to 5 s), and baselined to − 50–0 ms around de first tone (ST1) of the block. Trials exceeding ± 80 μV were rejected to remove artifacts in the signal (Int1 = 18.78 ± 1.24, Int2 = 18.62 ± 1.52, Int3 = 18.08 ± 1.74). Subjects with less than one-third of the trials in any of the conditions were excluded from the analysis (3 subjects, final number of subjects n = 37). AEPs were calculated with the FieldTrip software (Oostenveld et al. 2011) in a time window of −100 to 400 ms, through the *ft_timelockanalysis* function.

#### Functional near-infrared spectroscopy (fNIRS)

The continuous wave NIRScout device (NIRx Medical Technologies, Glen Head, NY, USA) was employed to obtain the hemodynamic fNIRS signal. An EASYCAP equipped with 14 LED sources and 17 detectors (avalanche photodiode, APD) positioned on the scalp, with 8 short channels and 32 standard channels, was used to record the changes in HbO and HbR (760 nm & 850 nm wavelength) concentrations in the auditory, visual and frontal cortices (Fig. 1b). The source-detector distance was 3 cm for the standard channels and 0.8 cm for short channels. Data acquisition was performed with NIRStar 14.2 software (NIRx Medical Technologies) at 4.46 Hz. The fNIRS Optodes'Location Decider software (fOLD; Zimeo Morais et al. 2018) was used to select the sources and detectors for the auditory, prefrontal, and visual cortices (Table 1). The Atlasviewer software (Aasted et al. 2015) was used to evaluate the sensitivity of the montage, this software allows us to observe whether the fNIRS optodes used cover the brain regions of interest and the degree of penetration (Fig. 1c).

**Table 1:** Standard fNIRS channels with the corresponding anatomical area following Brodmann’s areas

Right Hemisphere			Left Hemisphere		
Channel	BA	Brain area	Channel	BA	Brain area
01	22	STG-R	09	22	STG -L
02	22	MTG -R	10	22	MTG -L
04	22	STG -R	12	22	STG -L
05	22	STG -R	13	22	STG -L
06	40	SMG -R	14	40	SMG -L
07	39	AnG-R	15	39	AnG -L
19	17	Vsc -R	20	17	Vsc -L
21	9	SFG -R	29	9	SFG -L
22	9	SFG -R	31	9	MFG -L
23	45	IFG -R	32	45	IFG -L
24	9	MFG -R	34	9	SFG -L
25	45	IFG -R	35	9	SFG -L
27	9	SFG -R	36	45	IFG -L
37	47	IFG -R	39	47	IFG -L
38	45	IFG -R	40	45	IFG -L
28	9	Cingulum-Central	17	17	Vsc -Central

*BA* Brodmann area, *STG* superior temporal gyrus, *MTG* middle temporal gyrus, *SMG* supramarginal gyrus, *AnG* angular gyrus, *Vsc* visual cortex, *SFG* superior frontal gyrus, *MFG* middle frontal gyrus, *IFG* inferior frontal gyrus

The data pre-processing was performed in Homer2 (Huppert et al 2009) and MATLAB R2019b (MathWorks Inc., MA, USA). First, the noisy channels with extreme values (dRange: 0.03–2.5) or with a high standard deviation (SNR = 5 and coefficient of variation = 17) were removed using the *enPruneChannels* function (mean = 2.8 channels). Then, *hmrMotionCorrectionWavelet* with an IQR of 1.5, was applied to correct the motion artifacts in the data, through wavelet decomposition (Molavi and Dumont 2010; Brigadoi et al. 2014). *hmrMotionArtifact* and *enStimRejection* (tmotion = 0.5; tmask = 1.0, SDThresh = 15; AMPThresh = 0.7) were applied, with a time window of −2 to 10 s around the stimulus, to remove the trials with high standard deviation or higher amplitude. Considering the robustness of physiological artifacts in fNIRS data, especially in an auditory paradigm with high intensities (Muñoz et al. 2023), PCA was computed to extract the 2 first components of the data (*enPCAFilter;* nSV = 2). Finally, a regression of the short channels with the *hmrSSR* function was performed to extract the extracortical activity.

### Statistical analysis

#### EEG

**Fig. 2:**
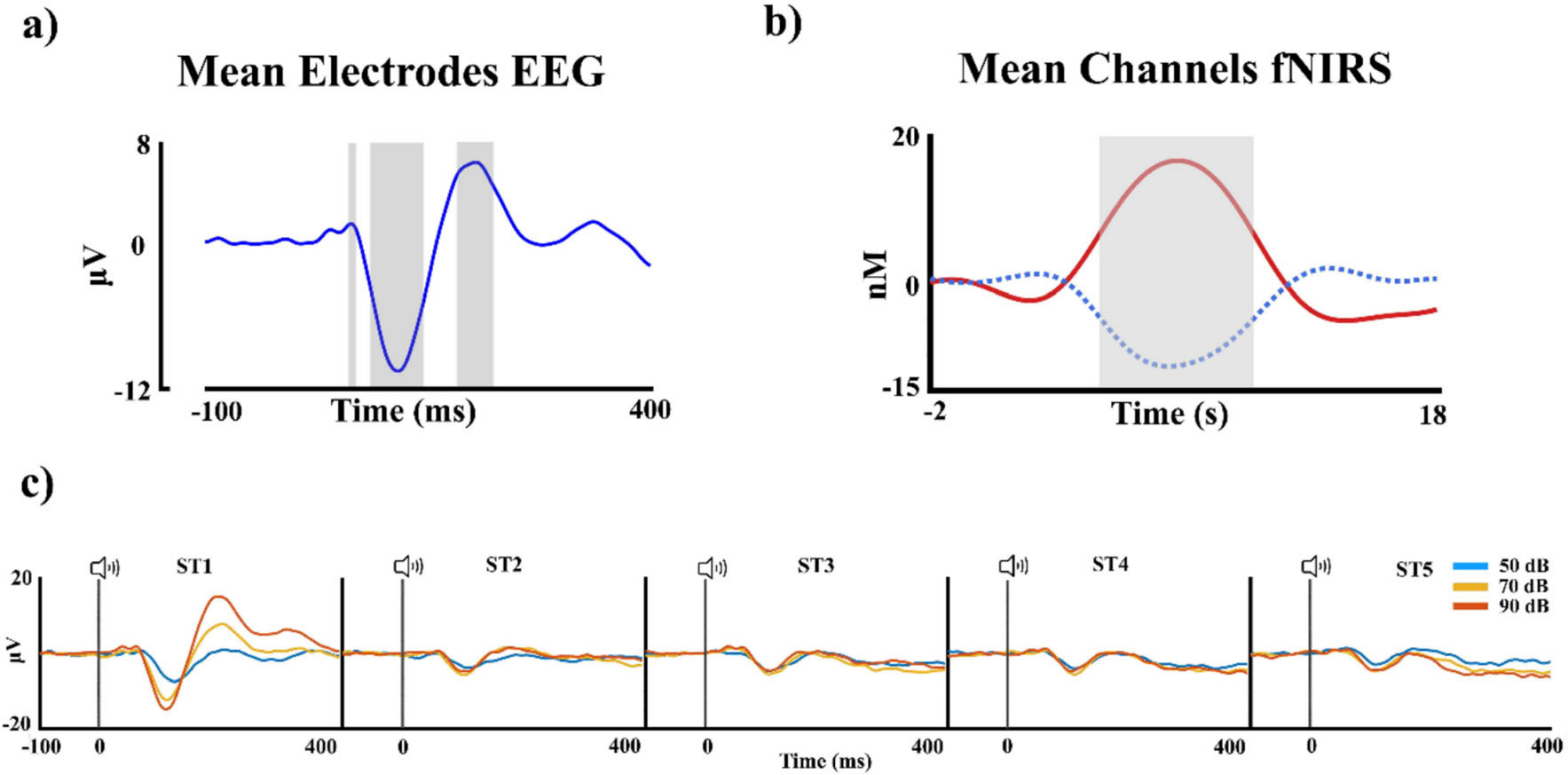
**a** Average of the electrodes and intensities in the EEG signal, to select the AEPs. **b** Average of the channels and intensities for the fNIRS channels, to select the time window. Each gray square shows the selected time window. **c** Event-related potentials at electrode Cz for the 5 tones delivered, showing a marked reduction in amplitude after the ST1

For the EEG signal, averaging across subjects and stimulus types was performed to select the time windows for AEP components in an unbiased manner (Fig. 2a) for the first stimulus of the block. The selected components were chosen by selecting the peak with the MATLAB functions *max* and *min* and then selecting the time points around the peak. The AEPs and time windows selected were P1 (58–68 ms), N1 (86–146 ms), and P2 (183–223 ms). The analysis of AEPs was restricted to responses to the first tone (ST1) within each block of tones, due to the sensory gating effects in auditory processing (Fig. 2c), whereby the amplitude of evoked potentials highly decreases with repeated stimuli within short interstimulus intervals. Statistical analysis for each AEP was performed using PERMANOVA in R with the *adonis2* function, this analysis approach uses permutation testing and therefore does not require normality in the data. The factors included were electrode and intensity, for each component (P1, N1, and P2). Finally, a post-hoc analysis was performed for the significant factors and corrected for multiple comparisons (false discovery rate: FDR; Benjamini and Hochberg 1995).

#### Functional near-infrared spectroscopy (fNIRS)

First, to check if the changes in HbO and HbR concentrations showed an amplitude dependence with the intensity similar to the AEPs approach, a block-average analysis was performed by ROI. The data of the *hmrBlockAverage* Homer2 function (− 2 to 18 s) were extracted through a MATLAB custom script, in which the data were averaged for all the channels and conditions to obtain, in an unbiased manner, the time window for the analysis as in the AEPs. Thus, using the *findpeaks* function, the peak of each hemodynamic signal (the *max* for HbO and the *min* for HbR) was extracted and averaged, selecting +/− 3 s around the mean peak, obtaining a time window of 4.6–10.6 s post-stimulus (Fig. 2b). The average fNIRS signal was extracted subject by subject for each stimulus, condition, and time window for ROI. For ROI selection, the FOLD software was employed, following the Brodmann areas for the auditory, visual, and prefrontal cortex, for a more restricted analysis the prefrontal cortex was divided into Brodmann area 9 (including superior frontal gyrus-SFG, middle frontal gyrus-MFG), and Brodmann area 45–47 (inferior frontal gyrus-IFG). Then, a two-way PERMANOVA analysis was performed for each chromophore with ROI and intensity as factors. The *p*-values of the post-hoc analysis for the significant effects were FDR-corrected. To analyze the latency of the hemodynamic response, which has been suggested to vary across cortical areas (Bauernfeind et al. 2018), only the ROIs with significant hemodynamic changes were considered. Thus, subject by subject and channel by channel, the peaks of the block average fNIRS signal were selected in the time window of 4.6–10.6 s, through the MATLAB function *max* and *min* (for HbO and HbR, respectively). Then, the values of the latency were averaged by ROI in a subject-by-subject manner and analyzed with a two-way PERMANOVA considering auditory intensity and ROI as factors. The post-hoc p-values were also FDR-corrected.

For the analysis of the topographies with a channel-by-channel approach, the extracted data preprocessed in Homer2 were imported into the NIRS-KIT software (Hou et al. 2021), where an individual General Linear Model (GLM) analysis was performed subject by subject for each independent channel. The GLM model considers the HbO and HbR signals as the dependent variables, which are defined by a linear combination of the conditions, in this case, the intensities and an error term. For GLM specification the software takes the canonical Hemodynamic Response function (HRF) response and provides the estimated best fit (betas) by subject for each condition, each channel, and for the specified contrasts. For contrast analysis 0, 1, and −1, were employed to compare the magnitude of the HbO or HbR signal between the conditions. Thus, the contrast vector [−1 1 0] is used to assign a weight of −1 to 50 dB intensity and a weight of 1 to 70 dB intensity. Similarly for the other comparisons: 50 dB vs 90 dB [−1 0 1] and 70 dB vs 90 dB [0 −1 1]. Since each contrast vector represents a different pairwise comparison between the intensities, they are naturally orthogonal to each other. To analyze the cortical activation channel by channel for each intensity, and the significant differences between them t-tests were run for each intensity individually, and for the specified contrast, the p-values were FDR-corrected through the group-level analysis in the NIRS-KIT software.

#### Correlation analysis

Finally, since one of the main objectives of the present report is to integrate the electrical and vascular information of both signals (EEG-fNIRS), a Spearman correlation analysis was conducted, with the residuals of the HbO, HbR, P1, and P2 variables to remove variance explained by their respective individual activity. Specifically, residuals were calculated by fitting linear models to the raw beta values for each signal, electrode, and channel, regressing out the global mean activity across intensities. This method aims to isolate stimulus-specific neural and vascular responses that are independent of global trends, which could be influenced by individual differences. Supplementary Fig. 1 illustrates this process, showing the broad inter-individual variations in all the analyzed signals. The rationale behind this approach is that neurovascular coupling can be obscured by global components, and removing these allows for a more direct examination of functional relationships between localized neural and hemodynamic responses. The betas of the HbR chromophore were multiplied by −1 to facilitate the interpretation of the correlations.

The residuals were calculated as follows:

1 $$Beta fNIRS Values\left( {ch} \right) = \beta 0 + Mean Beta values \left( {ch} \right) + rfNIRS$$

2 $$N1 = \beta 0 + Mean N1values \left( {ch} \right) + rN1$$

3 $$P2 = \beta 0 + Mean P2values \left( {ch} \right) + rP2$$

First, Specifically, for each fNIRS channel (Beta fNIRS Values(ch)), a linear regression model was fitted using the actual beta values from the GLM and the mean beta value (Mean Beta Values (ch)) across the three sound intensities for each subject (Supplementary Fig. 1; Eq. 1). The residuals from this model were extracted for further analysis (rfNIRS). Similarly, for AEPs, a linear model was applied to the N1 and P2 amplitudes for all the recorded electrodes (Supplementary Fig. 1; Eq. 2 and Eq. 3), and then the residuals were extracted (rN1; rP2). Before the calculation of the correlations, outliers in the data were detected and removed using the *isoutlier* function in MATLAB. Spearman correlations were then performed separately for HbO and HbR, including the residuals of AEP components each residual of the fNIRS standard channels, and the intensity. The correlations were performed in MATLAB with the *corr* function. Finally, all p-values were corrected by FDR. Significant correlations were visualized topographically to highlight the spatial distribution of neurovascular coupling effects across all recorded regions. Thus, following this approach to extract the residuals after accounting for their respective global trends, we aimed to uncover associations between neural and vascular activity that could have been hidden by individual differences.

## Results

**Fig. 3:**
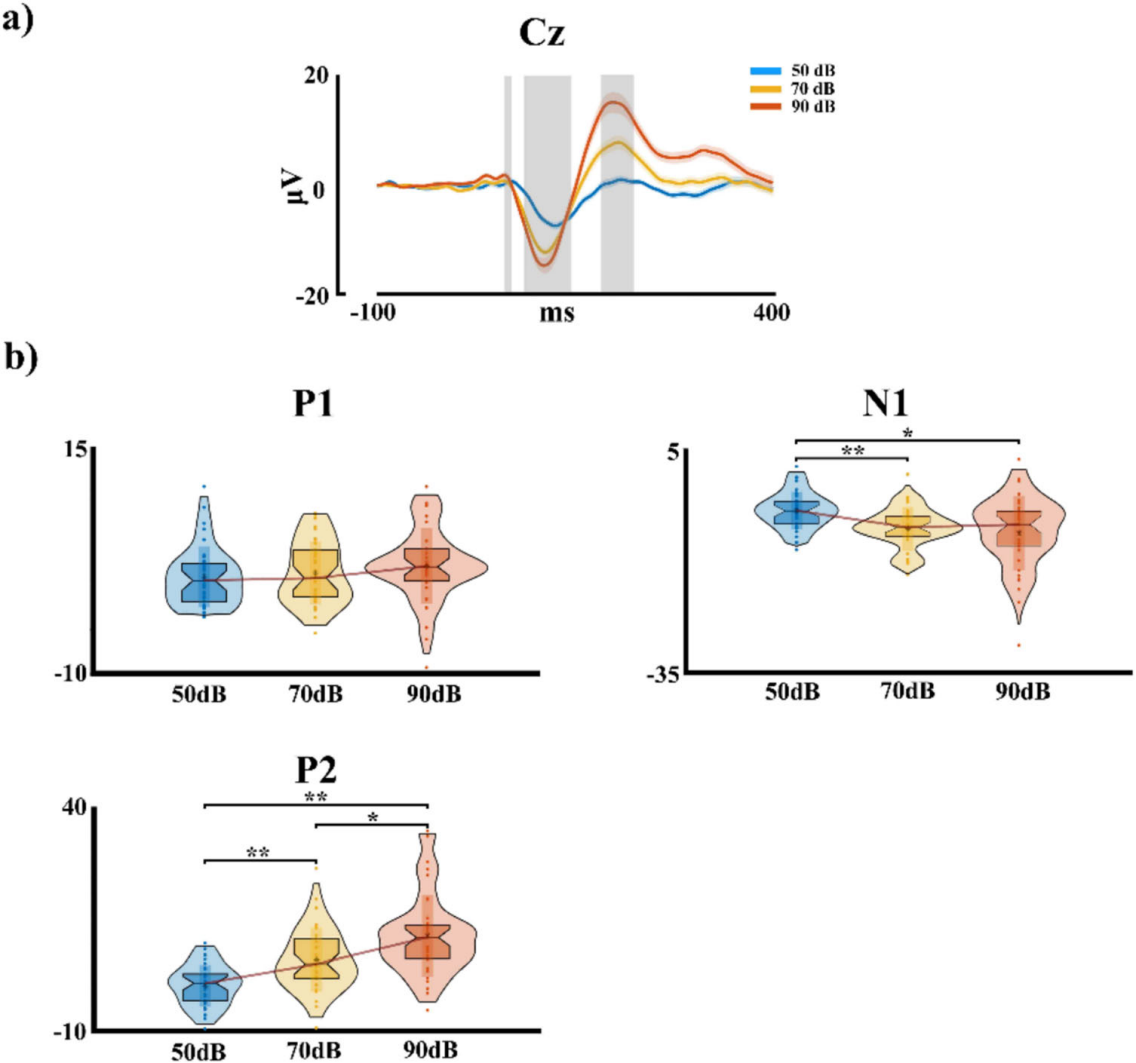
**a** Event-related potentials at electrode Cz showing the differential magnitude for each intensity presented. **b** Violin box plot for each of the event-related potentials showing the significant comparisons for each of them. The interception of the red line in the violin box plot coincides with the median. *p <.05 **p ≤.001

Figure 3a shows the intensity-dependent amplitude AEPs recorded at the Cz electrode for the three intensity levels. The intensity dependence seems to be particularly pronounced for the P2 AEP. Notably, the AEPs for the second to fifth tones in the sequence show a significant reduction compared to the first tone, likely due to sensory gating effects. Thus, the AEPs for the second to fifth tones (Fig. 2c) were not included in the statistical analysis, as they were beyond the scope of the present study. The statistical results of the PERMANOVA analysis for the P1 component show an effect of intensity (*pseudo*-F = 7.18, η2 =.016, *p* =.012). However, the post-hoc analysis between the intensities did not show significant differences. The N1 shows an effect for the intensity factor (*pseudo*-F = 46.90, η2 =.096, *p* =.001). The post-hoc analysis for the intensity shows differences between the intensity 1 (50 dB) with the intensities 2 (70 dB) and 3 (90 dB) (*p* <.001, Int2 > Int1; *p* =.003, Int3 > Int1, here > implies higher negativity). For the P2 component, the PERMANOVA shows an effect for the intensity factor (*pseudo*-F = 169.27, η2 =.276, *p* =.001). The post-hoc analysis shows significant differences between the intensity 1 (50 dB) with the intensities 2 (70 dB) and 3 (90 dB) (*p* <.001, Int1 < Int2; *p* <.001, Int1 < Int3), and for the intensity 2 (70 dB) with the intensity 3 (90 dB) (*p* =.006, Int2 < Int3). All multiple comparisons were corrected by FDR. The violin box plots for the intensity results in each AEP are shown in Fig. 3b.

**Fig. 4:**
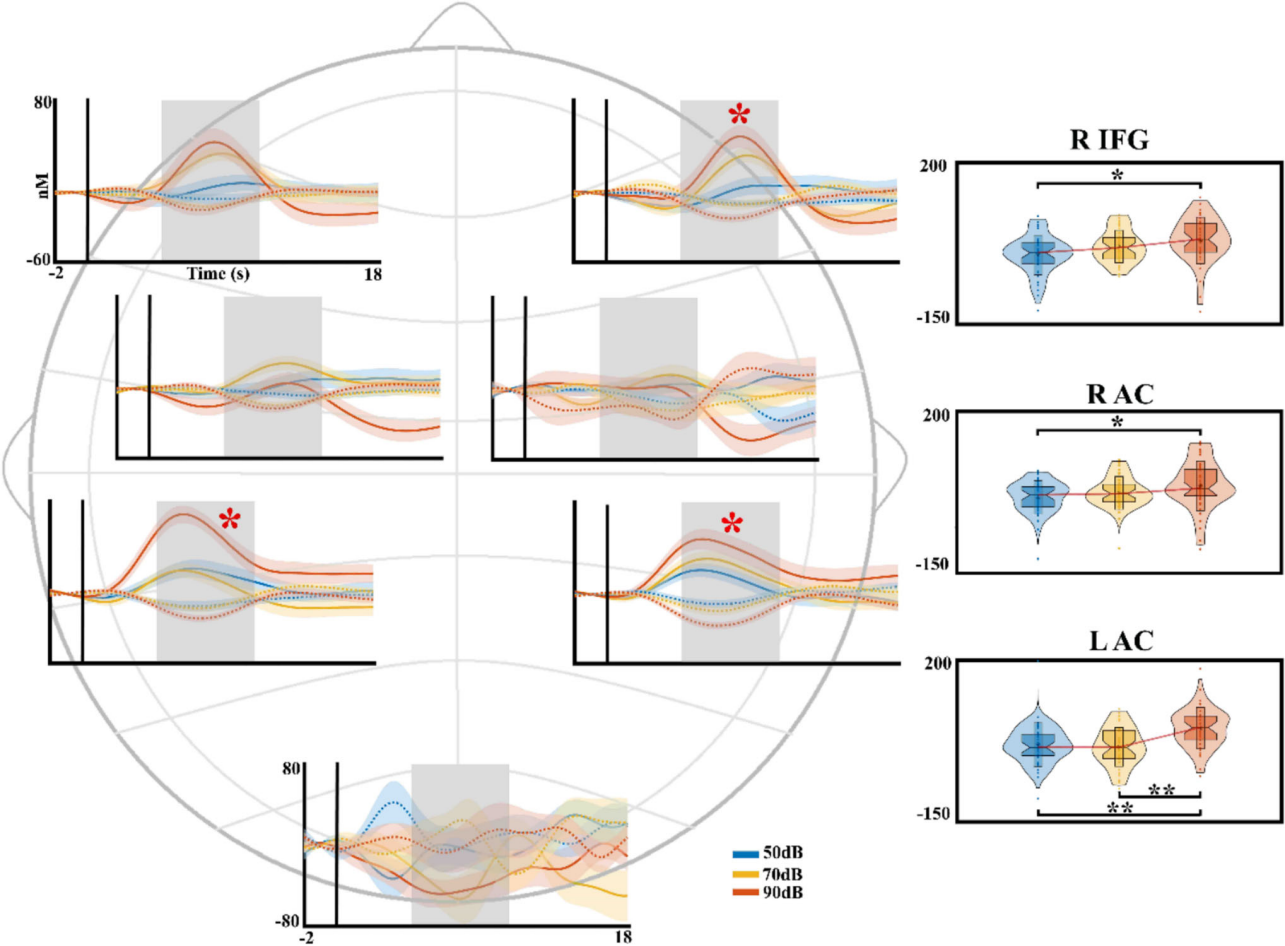
Block Average of the fNIRS signal by regions of interest (ROIs). The significant post-hoc interaction in the PERMANOVA is plotted next to the average figure for each ROI in a violin boxplot. The dotted lines indicated the changes in the deoxygenated hemoglobin. The red asterisk represents the ROIs with the effect of intensity in the PERMANOVA results. The red line in the violin boxplots represents the median. *p <.05 **p ≤.001. R IFG: Right Inferior Frontal Gyrus; R AC: Right auditory cortex; L AC: Left auditory cortex

Figure 4 shows the amplitude dependence of intensity for the different ROIs recorded. Although block averages are not the primary focus of this report, this analysis was conducted to determine whether the fNIRS response exhibits a dependence on the intensity of auditory stimuli, similar to the AEPs. Additionally, it allows for the computation of individual fNIRS peak latencies (see below). The block-average fNIRS PERMANOVA shows an effect of ROI (*pseudo*-F = 13.61, η2 =.087, *p* <.001) and intensity*ROI (*pseudo*-F = 2.80, η2 =.036, *p* <.001) for HbO. The post-hoc analysis for the interaction intensity*ROI shows a difference in the right auditory cortex between the intensities 1 (50 dB) and 3 (90 dB) (*p* =.012, Int1 < Int3), in the left auditory cortex between the intensities 1(50 dB) and 2 (70 dB) with the intensity 3 (90 dB) (*p* <.001, Int1 < Int3; *p* <.001; Int2 < Int3), and in the right IFG between the intensities 1 (50 dB) and 3 (90 dB) (*p* =.006; Int1 < Int3), for the left IFG a trend for significance after FDR correction between the intensity 1 (50 dB) with the intensities 2 (70 dB) and 3 (90 dB) was found (*p* =.055, Int1 < Int3; *p* =.055, Int1 < Int2). The HbR chromophore has no effect for the analyzed factors.

For the latency analysis of the block-averaged fNIRS signal the PERMANOVA shows an effect of ROI (*pseudo*-F = 18.20, η2 =.102, *p* <.001) in HbO. The post-hoc analysis shows significative differences between the right auditory cortex and the right and left IFG (*p* <.001, R AC < R IFG dif = −0.85 s; *p* <.001, R AC < L IFG, dif = −0.92 s), and between the left auditory cortex with the right and left IFG (*p* <.001, L AC < R IFG, dif = −0.77 s; *p* =.001, L AC < L IFG, dif = −0.84 s). For the HbR the PERMANOVA also shows an effect of ROI (*pseudo*-F = 6.79, η2 =.041, *p* <.001). The post-hoc analysis shows differences between the right auditory cortex with the right IFG (*p* =.002, R AC < R IFG, dif = −0.83 s) and the left auditory cortex with the right IFG (*p* =.034, L AC < R IFG, dif = −0.51 s). The violin box plots for the significative post-hoc analysis of the latency differences are displayed in Supplementary Fig. 2.

**Fig. 5:**
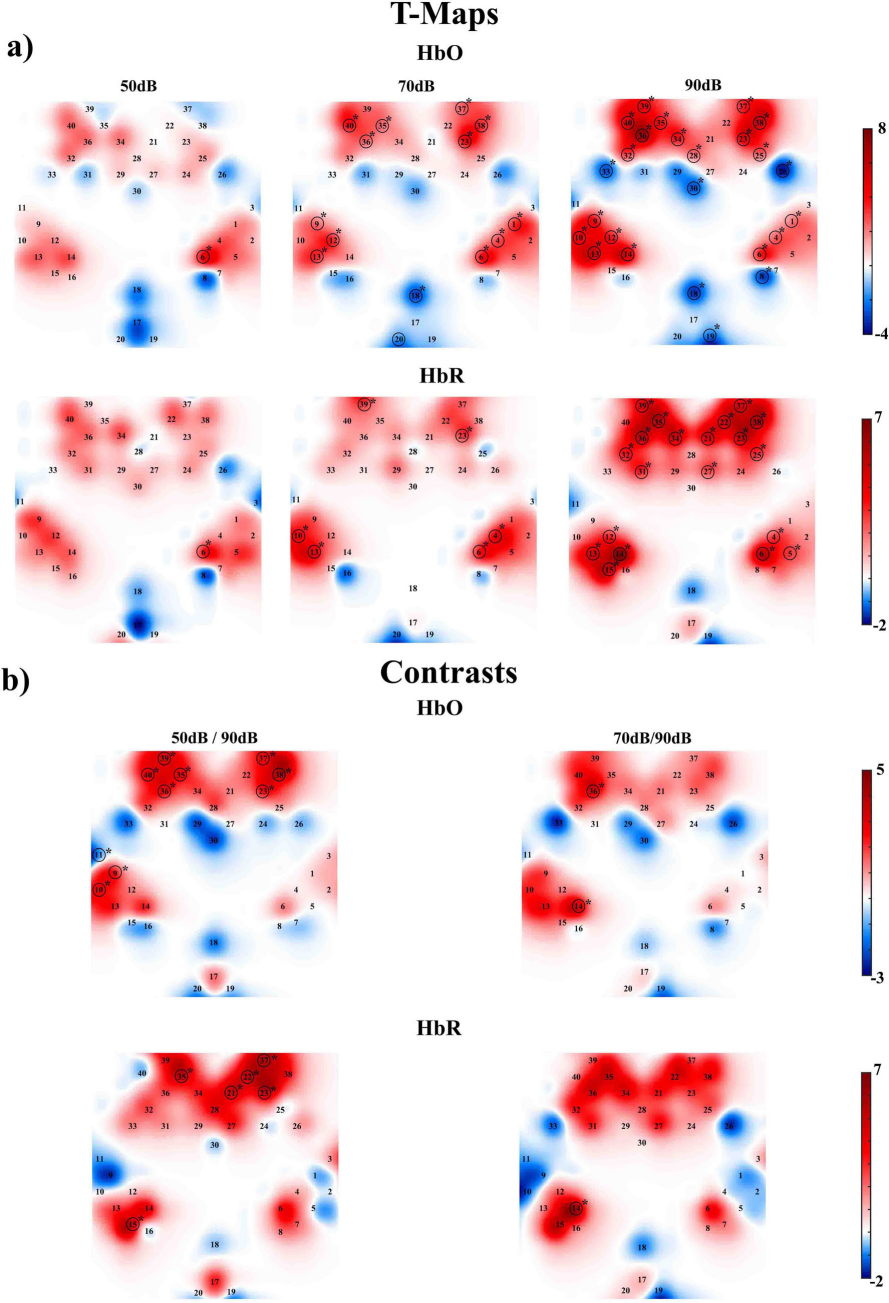
T-contrast map, for each of the **a** intensities and **b** their significant contrasts, for HbO and HbR. The significant channels are indicated with a circle and an asterisk (FDR corrected)

Figure 5a shows the T-maps of the beta values for each intensity and chromophore. As intensity increased, a greater number of frontal channels were activated. The FDR-corrected *p*-values for the significant channels are provided in Supplementary Table 1. Figure 5b shows the contrast analysis between the intensities. The results revealed no significant differences between 50 and 70 dB for either HbO or HbR. However, significant differences were found between both lower intensities (50 and 70 dB) and the highest intensity (90 dB) in both chromophores. For HbO, the intensities 2 (70 dB) and 3 (90 dB) showed significant differences in the auditory channel 14 (*p* = 0.019, Int2 < Int3) and prefrontal channel 36 (*p* = 0.019, Int2 < Int3); for the intensities 1 (50 dB) and 3 (70 dB) the significant differences were found in the auditory channels 9 (*p* =.042, Int1 < Int3), and 10 (*p* =.044, Int1 < Int3), in the prefrontal channels 23 (*p* =.036, Int1 < Int3), 35 (*p* =.036, Int1 < Int3), 36 (*p* =.035, Int1 < Int3), 37 (*p* =.036, Int1 < Int3), 38 (*p* =.005, Int1 < Int3), 39 (*p* =.035, Int1 < Int3), 40 (*p* =.044, Int1 < Int3), and the short channel 11 (*p* =.044, Int1 > Int3). For HbR the intensities 2 (70 dB) and 3 (90 dB) showed significant differences only in the auditory channel 14 (*p* =.031, Int3 > Int2), however, for the intensities 1 (50 dB) and 3 (70 dB) the significant differences were found in the auditory channel 15 (*p* =.021, Int3 > Int1) and in the prefrontal channels 21 (*p* =.038, Int3 > Int1), 22 (*p* =.021, Int3 > Int1), 23 (*p* =.021, Int3 > Int1), 35 (*p* =.021, Int3 > Int1), and 37 (*p* =.021, Int3 > Int1).

**Fig. 6:**
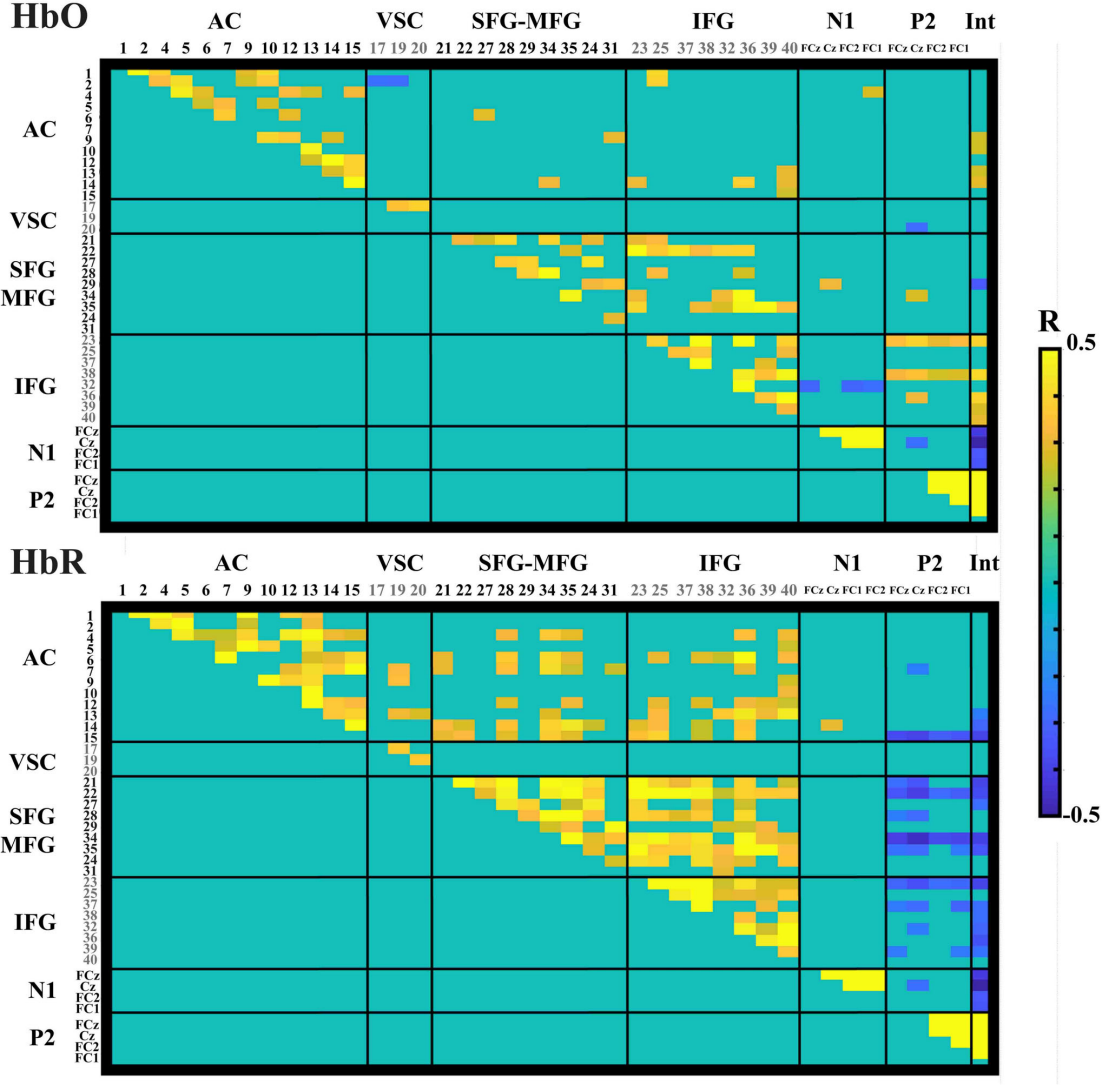
Spearman correlation matrix for the residuals of AEPs and the residuals of fNIRS betas. Only correlations that remained significant after false discovery rate (FDR) correction are shown. AC: Auditory cortex; VSC: Visual cortex; SFG: Superior frontal gyrus; MFG: Medial frontal gyrus; IFG: Inferior frontal gyrus; Int: Intensity

To address the understanding of neurovascular coupling, Spearman correlation analyses were performed on the residuals not explained by the global activity driven by individual differences for HbO and HbR, independently. The correlations show several significant correlations between AEP components and fNIRS hemodynamic activity (Fig. 6). For N1 the HbO showed positive correlation with one right auditory channel near the STG (Ch04: FC1, *p* =.030), and one channel in the SFG (Ch29: Cz *p* =.009), but negative associations with one IFG channels (Ch32: FCz *p* =.015, FC2 *p* =.019, FC1 *p* =.038). For HBR N1 showed positive correlations with one left auditory channel near SMG (ch14: Cz *p* =.008).

**Fig. 7:**
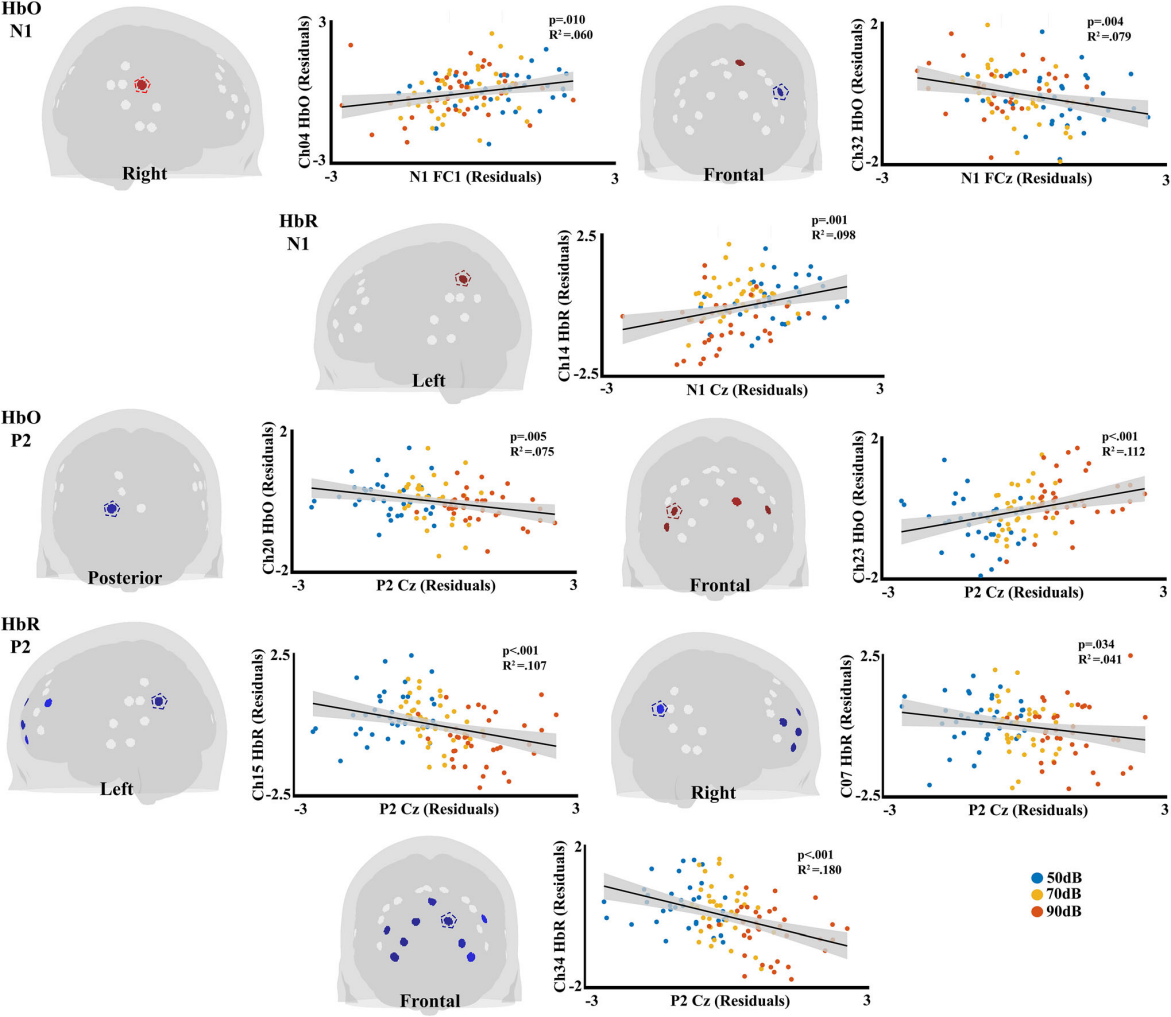
Representative linear regression plots for selected channels illustrating the relationship between auditory evoked potential residuals and fNIRS hemodynamic residuals. The topographies highlight the Spearman Correlation significant fNIRS channels for all electrodes associated with N1 and P2 in both HbO and HbR. Channels in blue on the topographies indicate negative correlations, while red channels indicate positive correlations. Channels marked with dashed lines are those plotted in the regression

The correlation for P2 in the HbO chromophore showed a negative correlation in one visual channel (ch20: Cz *p* =.027), and positive correlations in frontal regions with one SFG channel (Ch34: Cz *p* =.026), and three IFG channels (Ch23: FCz *p* =.004, Cz *p* <.001, FC2 *p* =.017, FC1 *p* =.005; Ch38: FCz *p* =.007, Cz *p* =.002, FC2 *p* =.045, FC1 *p* =.028; Ch36: Cz *p* = 0.008). For HbR, negative correlations were found with two auditory channels located more posteriorly, near the SMG and AnG (Ch7: Cz *p* =.042; Ch15: FCz *p* <.001, Cz *p* <.001, FC2 *p* =.005, FC1 *p* =.002), as well as several frontal channels within the SFG (Ch21: FCz *p* =.016, Cz *p* =.002; Ch22: FCz *p* =.002, Cz *p* <.001, FC2 *p* =.012, FC1 *p* =.003; Ch28: FCz *p* =.047, Cz *p* =.014; Ch34: FCz *p* <.001, Cz *p* <.001, FC2 *p* < 0.001, FC1 *p* <.001; Ch 35: FCz *p* =.021, Cz *p* =.015, FC1 *p* =.038) and IFG channels (Ch23: FCz *p* =.008, Cz *p* =.001, FC2 *p* =.016, FC1 *p* =.011; Ch37: FCz* p* =.038, Cz *p* =.013, FC1 *p* =.009; Ch32: Cz *p* =.043; Ch39: FCz *p* =.037, FC1 *p* =.043). The topographical representation for N1 and P2 is shown in the Fig. 7.

The Spearman correlation also shows the relationship between the standard fNIRS channels and the AEPs with intensity. For HbO, a positive correlation with intensity is observed in the left auditory cortex and almost all channels in the IFG. Interestingly, only one channel, channel 29 of the SFG, shows a negative correlation with intensity, while also correlating positively with N1. For AEPs, all electrodes show negative correlations with intensity for N1 and positive correlations for P2. For HbR, the expected negative correlations with intensity were found in the left auditory cortex, SFG, and IFG. It is well known that the HbR chromophore is less affected by physiological artifacts (Tachtsidis and Scholkmann 2016), which explains the increased number of observed relationships in this chromophore. Similar to HbO, all electrodes for AEPs correlate positively with intensity for N1 and negatively for P2.

In addition, Spearman correlation analysis also included the internal associations between standard fNIRS channels (Fig. 6). The results revealed several positive associations within the same brain regions, with auditory channels linking positively with other auditory channels and frontal channels showing positive connections within both the IFG and SFG channels in both HbO and HbR chromophores. Auditory channels also show positive associations with prefrontal channels in the SFG and IFG, suggesting functional connectivity between these regions, but this trend was more pronounced in the HbR chromophore. In contrast, a negative correlation was found between two visual channels and one specific auditory channel in the right middle temporal gyrus in HbO (Ch02-Ch17: *p* =.0.042, Ch02-Ch19: *p* =.034). Interestingly in HbR, the correlation between the visual cortex with auditory channels correlated positively but with auditory channels near STG and AnG (Ch07-Ch19: *p* =.005, Ch09-Ch19: *p* =.003, Ch13-Ch19: *p* =.011, Ch13-20: *p* =.025).

## Discussion

The results of the present report show an effect of amplitude dependence of AEPs, with increasing stimulus intensity leading to larger N1 and P2 components, but not affecting P1. Similarly, intensity-dependent changes in hemodynamic activity were observed in both the auditory and prefrontal cortices, as reflected in block average and GLM analysis. These changes exhibited a spatial distribution that expanded with increasing intensity, as shown in the T-maps, and are consistent with patterns reported in previous fMRI studies. Notably, prefrontal fNIRS activity in the IFG displayed a delayed response relative to the auditory cortex, suggesting distinct temporal dynamics between sensory and higher-order regions. Spearman correlations show auditory and prefrontal positive associations, suggesting functional connectivity between these areas. For the neurovascular coupling results, Spearman correlation of AEPs and fNIRS residuals suggested functional relationships between frontal and auditory regions, for N1 and P2. N1 showed positive associations with channels in the STG and SFG but negative associations with the IFG for HbO, while HbR positive correlation was found near the SMG. P2 correlations were predominantly observed in frontal regions, with positive associations in the SFG and IFG for HbO and negative associations with SMG, AnG, SFG, and IFG for HbR. Together these findings provide insights into the complex mechanism underlying auditory processing and cognitive engagement in response to varying sound intensities, and the electrical and vascular responses that are involved.

### AEPs

The effect of the intensity on the amplitudes of the P2 and N1 AEPs was found in the present report, indicating that AEP amplitudes increase with stimulus intensity (50 dB, 70 dB, and 90 dB). This finding is consistent with previous studies (Hegerl and Juckel 1993; Hegerl et al. 2001), which also reported the amplitude dependence of intensity. The modulation of AEP amplitudes by intensity can be attributed to increased neural synchrony and/or enhanced neural recruitment at higher intensities, in part modulated by serotonergic innervations. It has been proposed that the serotonergic effect on the auditory cortex plays a critical role in amplifying/reducing the AEPs, where high serotonergic neurotransmission would result in a weak IDAP and low serotonergic neurotransmission would result in a strong IDAP (Hegerl et al. 2001). In the present report, since the population studied were healthy volunteers without any neurological disorder, the expected modulation in AEPs was found. The effect of the intensity on the AEPs has primarily been associated with areas exhibiting higher serotonergic innervations, such as the auditory cortex. Additionally, the involvement of prefrontal regions, including the OFC, has been suggested (Hegerl and Juckel 1993; Hegerl et al. 2001; Mavrogiorgou et al. 2017). The participation of these areas is further supported by the results that will be discussed in the following paragraphs.

### Hemodynamic response

The hemodynamic results from both the block average and GLM-derived beta analyses demonstrate an amplitude-dependent response in the auditory and prefrontal regions as stimulus intensity increases. These results are in line with the fMRI and fNIRS studies which have found an intensity effect not only in the auditory area but also in the dorsolateral prefrontal cortex and OFC (Hart et al. 2003; Neuner et al. 2014; Bauernfeind et al. 2018; Muñoz et al. 2023). The fNIRS hemodynamic response, as observed in the block average, exhibits amplitude modulation like the AEPs, particularly in the auditory cortex and the right IFG, with a similar trend observed in the left IFG. These findings are consistent with those reported in our previous study (Muñoz et al. 2023). Furthermore, the present study investigates the effect of latency, revealing significant differences in response latency across ROIs, ranging from 510 to 920 m, with earlier latencies observed in the auditory cortex and later latencies in the IFG. These latency differences have been linked to delayed neural activation, network dynamics including less synchronous inputs to higher-order areas, and even physiological differences in vasculature or blood flow (Bauernfeind et al. 2018). In this context, the observed latency differences could be reflecting the dynamic flow of information between these regions. Rapid auditory processing in the auditory cortex facilitates swift responses to sound stimuli (Ogawa et al. 2007), while higher-order cognitive processes, such as attentional modulation, contribute to delayed hemodynamic responses in the IFG due to the engagement of more complex neural networks. These latency patterns align with literature suggesting hierarchical information transfer from auditory sensory to frontal areas in animals and humans (Planke and Rommanski, 2014, de Heer et al. 2017; Braga et al., 2017; Nourski et al. 2018, Jang and Choi 2022; Hockley and Malmierca 2024), supporting the idea that slower responses in frontal cortices would reflect integrative processing and top-down modulation.

The T-maps of the beta values indicate that the number of activated channels increases with sound intensity, extending to broader areas within the auditory and prefrontal cortices at 70 and 90 dB. This expansion in the cortical activation with intensity is consistent with previous findings by Hall et al., (2001) and Neuner et al. (2014), who reported that higher intensities recruit more extensive cortical and subcortical regions. This phenomenon likely reflects greater excitation within the auditory and prefrontal cortices, aligning with fMRI studies that show heightened activation in these areas as sound levels increase (Hall et al. 2001; Hart et al. 2003). Similarly, the contrast analysis (Fig. 5b) shows the differential topographical activation when comparing the lower intensities (50 dB and 70 dB) with the highest intensity (90 dB), with pronounced differences near the IFG, and auditory-related structures near the STG, SMG and AnG. According to Jäncke et al., (1998) the network between the STG and the IFG, has been associated with the retrieval and rehearsal of auditory information, and in the present study, this network would be specifically related to encoding high-intensity auditory stimuli. The last statement would be supported by the Spearman correlation results in which positive associations were found in a series of auditory and prefrontal channels, suggesting a coupling of auditory processing with higher-order cognitive functions. These findings would indicate a complex interplay between sensory and cognitive networks, with increasing auditory stimulus intensity potentially enhancing the connectivity between auditory and prefrontal regions, which may support the process of attention and cognitive control during auditory processing (Bidet-Caulet et al. 2015; Panichello and Buschman 2021).

Moreover, the internal fNIRS correlations reveal negative and positive correlations between auditory and visual channels in HbO and HbR, respectively. These seemingly contradictory results can be explained by the physiological properties of the oxygenated and deoxygenated hemoglobin. In HbO the total blood flow is distributed prioritizing certain regions, it is possible that with greater activation in the auditory channel (by increasing the intensity of the stimulus), more oxygen is captured in that region, while in the visual channel, the HbO does not increase in the same proportion. As the brain reallocates resources to enhance auditory processing during high-intensity stimuli, the reduced HbO in the visual domain can lead to an accumulation of deoxyhemoglobin, as the oxygen supply does not increase in the same proportion as metabolic demand (Pinti et al. 2020). This dynamic is particularly relevant in tasks involving multisensory stimulation, where sensory modalities compete for limited cognitive resources. In the present study, participants were engaged in watching a mute movie while auditory stimuli were delivered, highlighting the competition between sensory channels.

Additionally, the intra-regional positive correlations found in the Spearman Correlation would suggest an influence of regional cortical blood flow, this could imply that beyond neurovascular coupling, local vascular dynamics would contribute to the hemodynamic response. In this regard, it has been suggested that hemodynamic changes are driven by a complex interaction of cerebral blood flow (CBF), cerebral blood volume (CBV), the cerebral metabolic rate of oxygen (CMRO_2_), and vascular density, thus, this interaction would play an important role in the amplitude, and temporality (as discussed before) of the HRF (Kim and Ogawa 2012; Vigneau-Roy et al. 2014). Frontal areas, for instance, have been proposed to have a higher vascular density (Vigneau-Roy et al. 2014), therefore differences in neurovascular coupling efficiency could emerge from these variables, potentially complicating the interpretation of fNIRS signals in terms of neuronal activity alone.

### Neurovascular coupling

Source analyses of AEPs have shown that N1 and P2 typically follow a frontocentral scalp distribution (Näätänen and Picton 1987; Alcaini et al. 1994; Hyde 1997). Consistent with these findings, Spearman correlation analysis of residuals in the present study revealed significant correlations for the N1 component, specifically involving two auditory channels near the STG and SMG, as well as two frontal channels near the SFG and IFG. Notably, for HbO the auditory and SFG channels showed positive correlations whereas the IFG channel exhibited negative correlations. Physiologically, the N1 amplitude is associated with the processes of detection and attention triggering (Hyde 1997). Accordingly, the positive correlations observed in N1 amplitude (i.e., more negative values) with the STG and SFG channels (decrease in HbO) would reflect a localized reduction in metabolic demand, potentially influenced by attentional reallocation driven by sensory gating mechanisms. These processes are particularly relevant within the context of the current experimental design, which involves the presentation of tone sequences in blocks. Although sensory gating is not analyzed in the present study, it can be inferred from the observed reduction in amplitude of the potentials, as can be seen in Fig. 2c. Sensory gating or repetition suppression is a fundamental neural process that filters out redundant or irrelevant stimuli, allowing the brain to focus on salient sensory information (Freedman et al. 1996). In this context, the observed relationship suggests that early auditory processing (reflected in N1) is associated with the modulation of hemodynamic responses in frontal and auditory areas, thereby facilitating the suppression of irrelevant sensory input. Similarly, the negative correlation between N1 and HbO in IFG would suggest recruitment of the IFG for top-down control, increasing its metabolic demand. The IFG is known to play a critical role in switching attention and inhibitory processing (Aron et al. 2004; Hampshire et al. 2010), which aligns with the concept of filtering out irrelevant stimuli through sensory gating mechanisms.

Furthermore, the positive correlation between N1 and the left auditory channel of the SMG suggests an active process in this more posterior auditory area, consistent with an early auditory triggering, where the increase in amplitude of N1 was correlated with increased activity in this channel. Notably, this correlation in the left auditory cortex specifically for deoxygenated hemoglobin was also found in our previous study (Muñoz et al. 2023) and was linked to neurovascular coupling, understood as the dynamic interplay between neural activity and hemodynamic vascular responses, where electrical activity drives corresponding changes in cerebral blood flow to meet metabolic demands (Attwell et al 2010; Schei et al. 2012). Given that the N1 component is considered a marker of auditory processing, reflecting both the detection of auditory stimuli and the allocation of attentional resources (Näätänen and Picton 1987), the engagement of auditory and frontal sources would indicate that early sensory signals in the auditory cortex interact dynamically with the SFG and IFG, supporting a framework where sensory and cognitive processes are integrated to optimize perception.

In contrast, the P2 component shows correlations mainly with frontal activity for the HbO and HbR within IFG and SFG, and also posterior auditory channels near the SMG and AnG for HbR. The correlations were positive for HbO and negative for HbR across all regions, suggesting that an increase in amplitudes of the P2 potential is correlated with the increase in HbO values and decrease in HbR values, i.e., activation in these areas. This functional association of P2 with frontal activity aligns with our previous study (Muñoz et al. 2023). Currently, the sources for P2 are discussed, but functionally has been linked to attentional targeting, perceptual learning, and inhibitory processes for irrelevant stimuli (Paiva et al. 2016). In this sense, similar to N1, the involvement of the IFG and SFG, would be crucial for top-down control, facilitating the modulation of attention and inhibition of distractions (Aron et al. 2004; Hampshire et al. 2010; Wegrzyn et al. 2017). The greater number of observed correlations of P2 with frontal activity (IFG and SFG) suggest a stronger involvement of top-down control mechanisms compared to N1. Thus, P2 would reflect the process of refining attention and integrating sensory information with cognitive demands, supporting sustained attentional engagement, and facilitating learning and adaptation to auditory stimuli. Although N1 and P2 are both related to frontal and auditory cortices, their distinct roles highlight two complementary but functionally distinct mechanisms of auditory-cognitive processing. N1 would reflect early sensory encoding and an early attentional capture, and P2 would reflect a later stage of attentional regulation, engaging both auditory and prefrontal regions in a broader network interaction. This distinction underscores the dynamic interplay between sensory and cognitive processes, where early sensory responses driven by the auditory cortex trigger top-down regulatory mechanisms.

A critical aspect of our analysis is the removal of the global response in each channel and electrode, to isolate stimulus-specific activity while preserving functionally relevant neurovascular coupling patterns. Is well known that the fNIRS signal has a lower signal-to-noise-ratio than fMRI, and is more contaminated by physiological artifacts (Cui et al. 2011; Tachtsidis, and Scholkmann 2016), in this sense some studies have tried to address this limitation (Zhang et al. 2016). In the present study, our approach is based on the premise that global trends in both EEG and fNIRS signals often reflect systemic noise, inter-individual variability, or non-neural contributions, which can obscure meaningful neural-vascular interactions. By extracting residuals, i.e., the variance that remains after accounting for these global trends, we aim to enhance the specificity of our analysis to activity directly linked to auditory stimulation. Importantly, the correlations observed in our study align with well-established neuroanatomical and functional relationships, particularly between auditory and prefrontal regions, further supporting the validity of the residual-based approach. Rather than distorting neurovascular coupling, removing the global response channel by channel and electrode by electrode seeks to refine the detection of functionally relevant interactions by minimizing individual confounds and allowing for a more precise interpretation of the relationship between electrophysiological activity and hemodynamic responses.

One limitation of the present study is the spatial discrepancy between EEG and fNIRS recordings, as EEG captures activity from central electrodes (FCz, FC1, FC2, Cz), although influenced by the volume conduction influences from other brain areas, while fNIRS optodes covered auditory, prefrontal, and visual cortices with a better spatial resolution than EEG. Despite this discrepancy, the observed correlations between residual AEPs and fNIRS signals provide insights into neurovascular coupling. Functional connectivity between auditory and prefrontal regions has been well-documented, with studies showing that higher-order cognitive processes, such as attention and auditory perception, engage distributed networks (Jäncke et al. 1998). Additionally, the residual-based approach used in this study helps isolate stimulus-specific relationships by reducing confounding effects from individual variations in AEP and fNIRS values. While future studies with high-density EEG and improved source localization techniques could refine the spatial alignment of EEG and fNIRS signals, the present findings offer evidence of functionally relevant neurovascular interactions, particularly in auditory and prefrontal cortices, where stimulus intensity effects were observed.

These findings contribute to an integrated, dual-modality perspective on auditory processing by combining EEG and fNIRS to examine how auditory stimulus intensity shapes both neural and vascular responses. This multimodal approach allows for a more comprehensive understanding of neurovascular coupling, capturing both the fast electrophysiological dynamics and the spatial distribution of hemodynamic activity. Specifically, the observed functional associations between auditory and prefrontal regions suggest that higher stimulus intensities not only engage auditory processing but also recruit non-auditory functions, such as attentional control and higher-order cognitive processes, which are mediated by the prefrontal cortex and are distinctly related to the N1 and P2 AEP functions. This interaction highlights the integration of sensory and cognitive systems in processing auditory stimuli. Given the extensive literature on IDAP in clinical population studies, this type of multimodal analysis could be applied to various disorders, such as ADHD or affective disorders, potentially aiding in targeting serotonergic pathways through non-invasive brain stimulation or pharmacological interventions. Limitations of the study are the penetration of fNIRS only at the cerebral cortex level, that most participants are right-handed, and that when co-recording the two signals the EEG noise-to-ratio and different confounds such as the use of tone blocks could alter the results.

## Conclusion

The results of the present report support models of auditory processing that emphasize the importance of intensity in sensory coding. In particular, the increase in AEP and hemodynamic response amplitude with sound intensity likely reflects neural synchrony and enhanced neural recruitment in auditory and prefrontal cortices, possibly indicating greater activation in these regions at higher sound levels. Similarly, these results suggest that the N1 and P2 components are related functionally with the auditory and prefrontal activity possibly involving early auditory encoding and cognitive resources such as attentional modulation and inhibitory processing. Taken together, these results suggest a link between auditory stimulus intensity and neural engagement that could have implications for understanding auditory perception in both healthy and clinical populations. Furthermore, the combined use of EEG and fNIRS provides a non-invasive, portable, and participant-friendly approach that is especially well-suited for clinical and sensitive populations. This dual-modality setup enables the simultaneous investigation of rapid neural dynamics and their associated vascular responses, offering a more comprehensive framework for assessing auditory function.

## Supplementary Information

Below is the link to the electronic supplementary material.

Supplementary file1 (DOCX 645 kb)

## Data Availability

The dataset generated and analyzed in the present study is available under reasonable request to the corresponding author (lmunnoz@us.es).
