# Supplementary material for: Sound intensity-dependent cortical activation: implications of the electrical and vascular activity on auditory intensity
Source: Cogn Neurodyn. 2025 Jun 9;19(1):88. doi: 10.1007/s11571-025-10281-7 (PMC12149058; doi:10.1007/s11571-025-10281-7)
Supplement: Supplementary file 1 — Supplementary file1 (DOCX 645 kb) [file 11571_2025_10281_MOESM1_ESM.docx]

**Supplementary material.**

| **Supplementary Table 1.** p-values FDR corrected of each intensity for the T-values fNIRS GLM | | | | | | | |
| --- | --- | --- | --- | --- | --- | --- | --- |
| **HbO** | | | | **HbR** | | | |
| **Intensity** | **Ch** | **Brain area** | **p** | **Intensity** | **Ch** | **Brain area** | **p** |
| 50db | 06 | SMG-R | <.001 | 50db | 06 | SMG-R | .022 |
| 70dB | 01 | STG-R | .004 | 70dB | 04 | STG-R | .001 |
|  | 04 | STG-R | .018 |  | 06 | SMG-R | .006 |
|  | 06 | SMG-R | .002 |  | 10 | MTG-L | .006 |
|  | 09 | STG-L | .046 |  | 13 | STG-L | .001 |
|  | 12 | STG-L | .004 |  | 23 | IFG-R | .030 |
|  | 13 | STG-L | .009 |  | 39 | IFG-L | .009 |
|  | 20 | VsC | .036 | 90dB | 04 | STG-R | .013 |
|  | 35 | SFG-L | .046 |  | 05 | STG-R | .046 |
|  | 23 | IFG-R | <.001 |  | 06 | SMG-R | <.001 |
|  | 37 | IFG-R | .027 |  | 12 | STG-L | .006 |
|  | 38 | IFG-R | .024 |  | 13 | STG-L | <.001 |
|  | 36 | IFG-L | .027 |  | 14 | STG-L | <.001 |
|  | 40 | IFG-L | .004 |  | 15 | AnG-L | <.001 |
| 90dB | 01 | STG-R | .026 |  | 21 | SFG-R | <.001 |
|  | 04 | STG-R | .013 |  | 22 | SFG-R | <.001 |
|  | 06 | SMG-R | .001 |  | 27 | SFG-R | .023 |
|  | 09 | STG-L | <.001 |  | 35 | SFG-L | <.001 |
|  | 10 | MTG-L | .001 |  | 34 | SFG-L | <.001 |
|  | 12 | STG-L | <.001 |  | 31 | MFG-R | .005 |
|  | 13 | STG-L | <.001 |  | 23 | IFG-R | <.001 |
|  | 14 | STG-L | <.001 |  | 25 | IFG-R | .018 |
|  | 19 | VsC | .002 |  | 37 | IFG-R | .001 |
|  | 28 | Cingulum-C | .025 |  | 38 | IFG-R | <.001 |
|  | 35 | SFG-L | .002 |  | 32 | IFG-L | .006 |
|  | 34 | SFG-L | .001 |  | 36 | IFG-L | <.001 |
|  | 23 | IFG-R | <.001 |  | 39 | IFG-L | <.001 |
|  | 25 | IFG-R | .020 |  |  |  |  |
|  | 37 | IFG-R | .002 |  |  |  |  |
|  | 38 | IFG-R | <.001 |  |  |  |  |
|  | 32 | IFG-L | .004 |  |  |  |  |
|  | 36 | IFG-L | <.001 |  |  |  |  |
|  | 39 | IFG-L | .001 |  |  |  |  |
|  | 40 | IFG-L | <.001 |  |  |  |  |
|  | 08 | ShCh | .011 |  |  |  |  |
|  | 18 | ShCh | .006 |  |  |  |  |
|  | 26 | ShCh | .002 |  |  |  |  |
|  | 30 | ShCh | .020 |  |  |  |  |
|  | 33 | ShCh | .018 |  |  |  |  |
| STG: Superior Temporal Gyrus, MTG: Middle Temporal Gyrus  SMG: Supramarginal gyrus, AnG: Angular Gyrus, Vsc: Visual Cortex,  SFG: Superior Frontal Gyrus, MFG: Middle Frontal Gyrus,  IFG: Inferior Frontal Gyrus, R:Right, L:Left, C:Central. | | | | | | | |


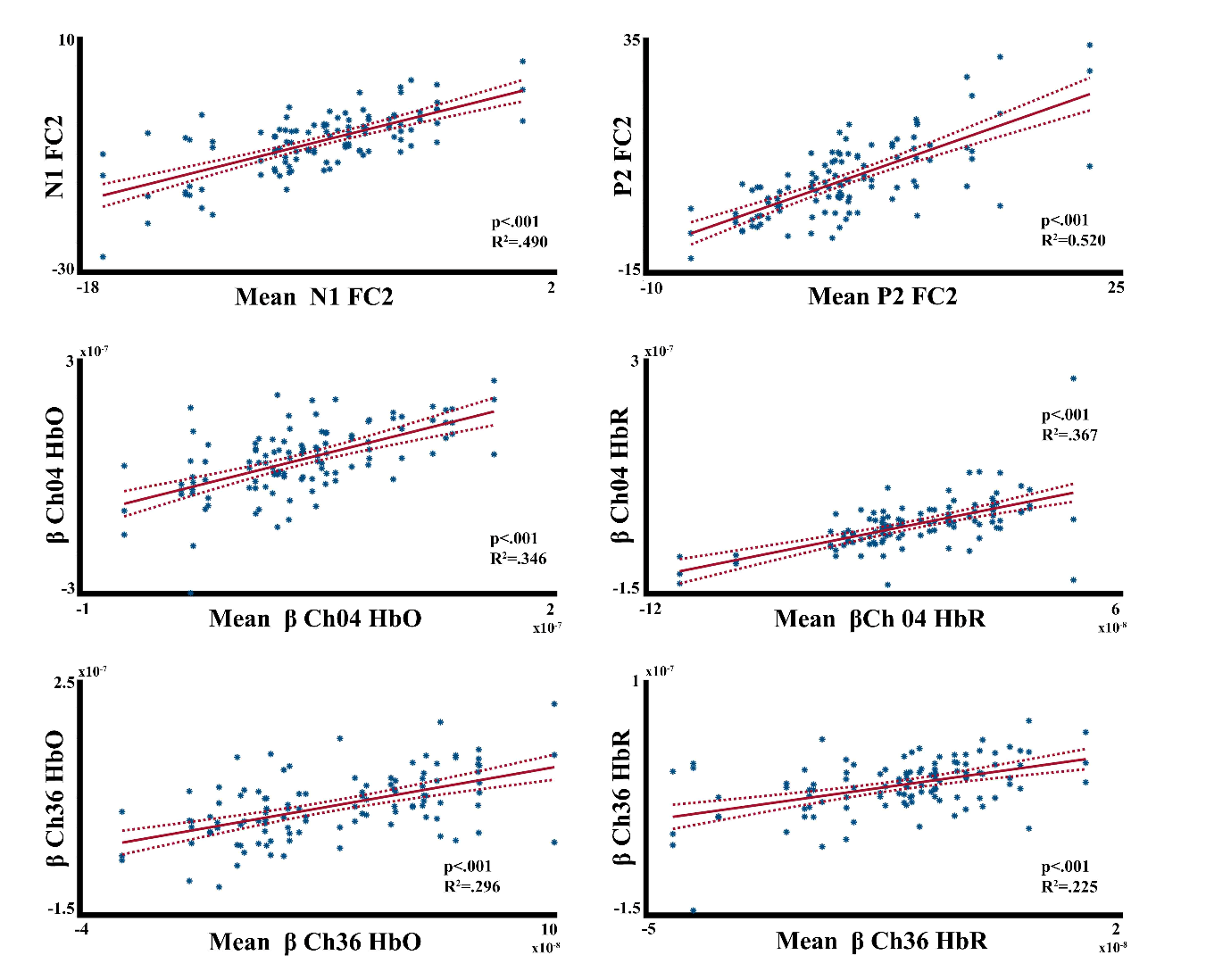


**Supplementary Fig 1.** Examples of the linear regression performed for the analyzed variables (N1, P2, fNIRS channels), to extract the residuals of these calculations


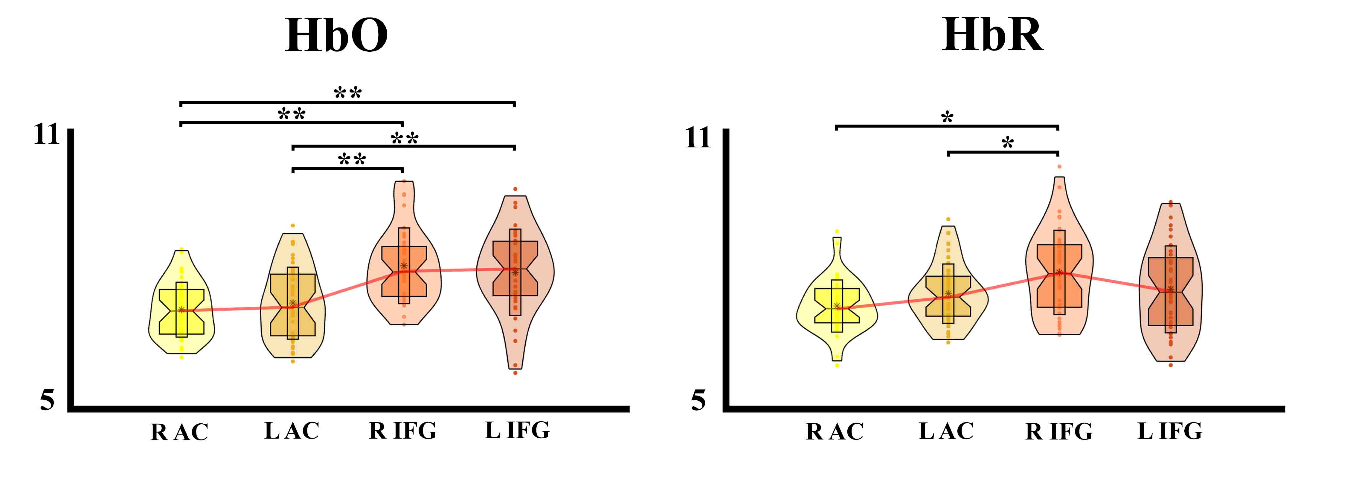


**Supplementary Fig. 2** Violin Boxplot of the significant post-hoc interaction in the PERMANOVA of the latency analysis in the block average fNIRS approach for HbO and HbR. The interception of the red line in the violin box plot coincides with the median. *p<.05 **p ≤.001. R AC: Right auditory cortex; L AC: Left auditory cortex: R IFG: Right Inferior Frontal Gyrus; L IFG: Left Inferior Frontal Gyrus
